# Differential Expressions of Adhesive Molecules and Proteases Define Mechanisms of Ovarian Tumor Cell Matrix Penetration/Invasion

**DOI:** 10.1371/journal.pone.0018872

**Published:** 2011-04-19

**Authors:** Youngjoo Kwon, Edna Cukierman, Andrew K. Godwin

**Affiliations:** 1 Department of Pathology and Laboratory Medicine, University of Kansas Medical Center, Kansas City, Kansas, United States of America; 2 Cancer Biology Program, Fox Chase Cancer Center, Philadelphia, Pennsylvania, United States of America; Cedars-Sinai Medical Center, United States of America

## Abstract

Epithelial ovarian cancer is an aggressive and deadly disease and understanding its invasion mechanisms is critical for its treatment. We sought to study the penetration/invasion of ovarian tumor cells into extracellular matrices (ECMs) using a fibroblast-derived three-dimensional (3D) culture model and time-lapse and confocal imaging. Twelve ovarian tumor cells were evaluated and classified into distinct groups based on their ECM remodeling phenotypes; those that degraded the ECM (represented by OVCAR5 cells) and those that did not (represented by OVCAR10 cells). Cells exhibiting a distinct ECM modifying behavior were also segregated by epithelial- or mesenchymal-like phenotypes and uPA or MMP-2/MMP-9 expression. The cells, which presented epithelial-like phenotypes, penetrated the ECM using proteases and maintained intact cell-cell interactions, while cells exhibiting mesenchymal phenotypes modified the matrices via Rho-associated serine/threonine kinase (ROCK) in the absence of apparent cell-cell interactions. Overall, this study demonstrates that different mechanisms of modifying matrices by ovarian tumor cells may reflect heterogeneity among tumors and emphasize the need to systematically assess these mechanisms to better design effective therapies.

## Introduction

Epithelial ovarian cancer (EOC), along with related Müllerian duct adenocarcinomas of the peritoneum and fallopian tube, are associated with the highest case/fatality ratio for all gynecologic malignancies diagnosed and is the fifth leading cause of cancer death in women in the U.S. [1]. Delay in diagnosing ovarian cancer is common, since the disease confined to the ovary seldom produces symptoms. As a result, the majority of cancers are diagnosed when the cancer involves one or both ovaries and is actively spreading beyond the pelvis to the lining of the abdomen and/or to adjacent lymph nodes [2], [3]. Therefore, understanding invasion strategies of ovarian cancer cells is important for the clinical management of ovarian cancer.

EOCs are considered to arise from the ovarian surface epithelium (OSE), a monolayer of cells that overlies the ovary and lines postovulatory inclusion cysts [4] or the fallopian tube in some hereditary cases [2]. Once an ovarian epithelial cell undergoes transformation, it detaches from the underlying matrix and can spread, often in clusters, by direct extension to adjacent organs [5]. Dissemination of EOC cells through the vasculature is generally rare, although the presence of metastases in extra-peritoneal sites (e.g., bone marrow, brain, and liver) has been reported in advanced-stage disease [6], [7], [8]. Ovarian tumor cells appear more likely to exfoliate and be transported by normal peritoneal fluid as multi-cellular aggregates [5], [9]. Exfoliated cells are implanted through discrete steps; adhesion to mesothelial cells, penetration or invasion throughout the peritoneal cavity, the omentum and the peritoneum [5]. The precise molecular mechanisms that control the penetrating invasion into the stroma and consequent dissemination to the peritoneum are unknown. Some studies suggest that the loss of E-cadherin expression could be involved in this process [9], [10] as tumor cells, including EOC cells, are often thought to undergo epithelial to mesenchymal transition (EMT) and invade as single cells through the stroma.

However, several lines of evidences suggest that EOC cells may invade using strategies other than the traditional EMT mechanism. First, more often than not, ovarian tumors are characterized by pathological criteria as invasive and malignant, yet they maintain E-cadherin expression [11], [12]. In addition, EOC and normal OSE are distinct from other epithelial cell-derived cancers and other normal epithelia, respectively. Remarkably, human normal OSE present both epithelial and mesenchymal phenotypes [4], [5] whereas they often lose mesenchymal characteristics and increase E-cadherin protein levels as these normal epithelial cells become malignant [4], [9], [11], [13], [14]. Moreover, the relevance of traditional EMT as a major invasion mechanism *in vivo* has been challenged [15], [16]. Therefore, besides well-studied mesenchymal cell migration accompanied by EMT, ovarian cancer cells may invade through additional mechanisms.

Recent studies demonstrated that in the absence of EMT, many types of cancer cells can invade as single cells without the use of proteolysis (e.g., amoeboid cell migration) or as collective aggregates without losing their cell-cell interactions (e.g., collective cell migration as well as collective growth) [17]. In the collective cell migration strategy, cells move as groups consisting of multiple cells connected through cell-cell junctions [18], [19], [20]. This type of movement occurs *in vivo* during morphogenesis and wound repair [19]. Also, it has long been observed that biopsies in cancer patients often contain groups of cells which either maintain contact with primary site (protruding sheets or strands) or are detached from their origin (nests) [19], [21]. These collective cells are known to rely on proteolysis to move through ECMs [22], [23].

Different from proteolysis-dependent collective or mesenchymal single cell invasion, protease-independent amoeboid invasion mechanism has been described in cancer cells and sarcoma cells upon treatments with protease inhibitors [24], [25], [26]. It was also reported that many types of cancer cells, which do not express appreciable amount of ECM-degrading proteases, can invade using the amoeboid strategy [26], [27]. In amoeboid invasion, the up-regulation of Rho and Rho-associated serine/threonine kinase (ROCK) is considered to be responsible for the generation of actomyosin forces that allow rounded and blebbing cancer cells to contract matrices and push their cell bodies through ECM fibers [25], [26].

In this study, we evaluated how ovarian tumor cell lines penetrate or invade through ECM using a three-dimensional (3D) culture model to mimic stroma *in vivo* conditions [28], [29], [30]. We report that ovarian tumor cells invade the mesenchymal/connective tissue-like ECM using primarily two distinct strategies; some cells appear to degrade the ECM using collective cell migration mechanisms whereas others form less tight cell-cell interactions and invade through the ECM by rearrangement (or possible contraction) of the ECM substrate. Protein profiles of epithelial and mesenchymal cell markers helped defining the invasion mechanisms utilized by the various cells. Penetration/invasion of cells with epithelial cell characteristics (e.g., OVCAR5) was mainly repressed by protease inhibitors while invasion of cells which exhibited a more mesenchymal marker expression profile (e.g., OVCAR10) was suppressed by ROCK but not protease inhibitors. Our study suggests that characterization of the penetrating/invading strategy used by ovarian cancer cells is required to select adequate therapies that will effectively target ovarian cancer behavior.

## Results

### Ovarian tumor cells penetrate (i.e., invade) through ECMs using primarily two different mechanisms

**Figure 1:**
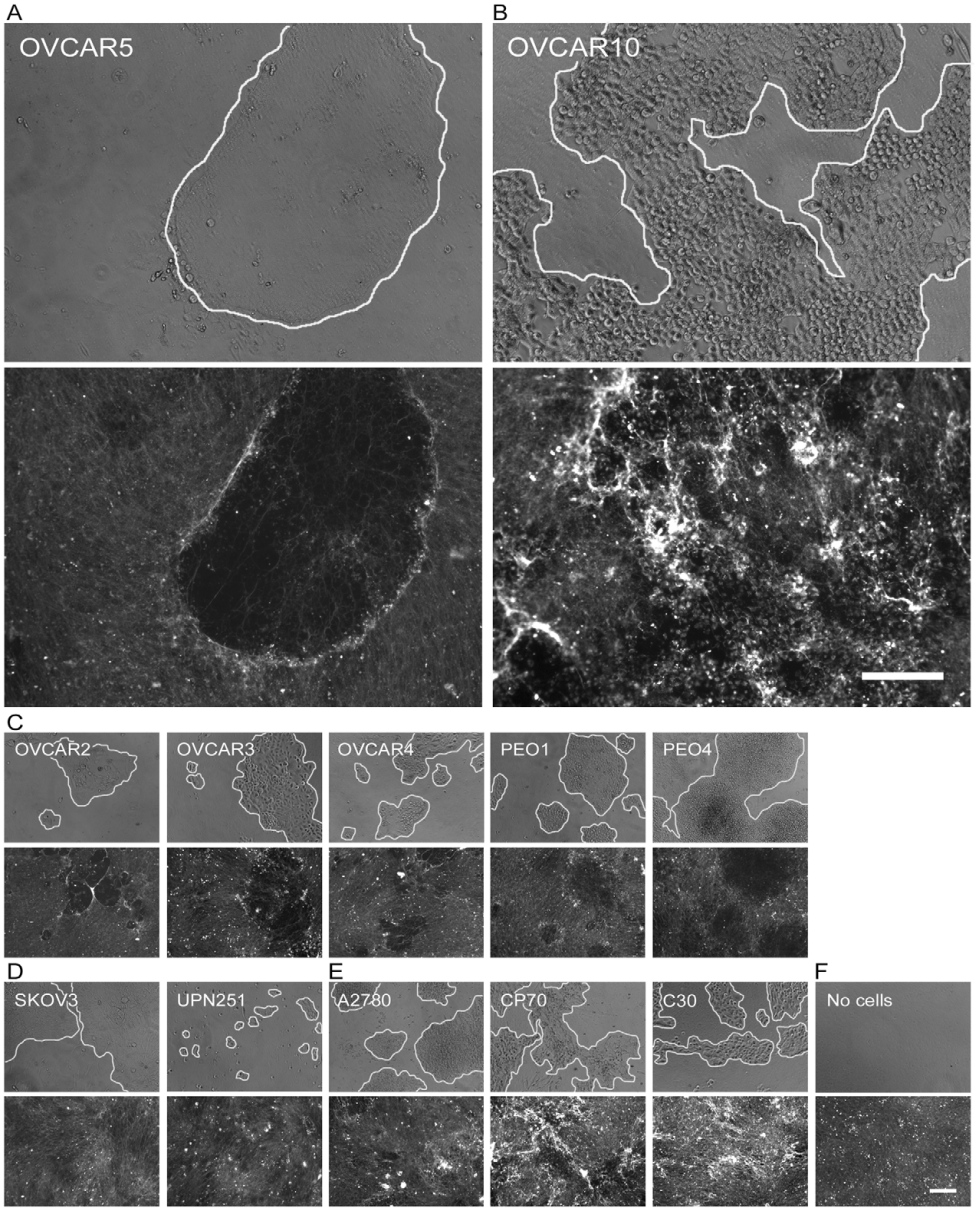
Differential ECM modifying mechanisms of ovarian tumor cells. A panel of ovarian tumor cells was plated onto pre-stained N3F-derived matrices and phase contrast (cells, top panel) and fluorescence (matrices, bottom panel) images were acquired over time during the culturing of cells. Depending on appearance of cell-cell interactions and ECM modifications induced during culturing cells within matrices, cells were divided largely into 3 different categories. OVCAR5 (A) and OVCAR10 (B) cells that represent the two primary ECM remodeling phenotypes are enlarged for the comparison. Some cells including OVCAR5 looked very tightly connected and their growth within ECMs resulted in degradation of the ECM (A & C). Other cells appeared to degrade the ECM while they organized in a single-cell configuration at early culturing times (D). Lastly, cells in the third category including OVCAR10 formed less tight cell-cell interactions than cells grouped with OVCAR5 while ECMs appeared to be rearranged, accumulated, or contracted (B & E). Matrices maintained without cells did not undergo remodeling (F). Images shown are representatives of each cell line and its corresponding ECMs after 10 days of culturing. Bar represents 200 µm.

We used a well studied fibroblast-derived 3D culture model to examine how various ovarian tumor cells penetrate or invade through an underlying *in vivo*-like 3D ECM [28], [29], [30], [31]. NIH-3T3 fibroblast (N3F)-derived matrices were used for the majority of analyses since this cell line reproducibly produced uniformly thick ECMs. Similar results were produced using matrices derived from tumor-associated fibroblast (TAFs, see Materials and Methods for details) in pilot studies (limited data are shown below). Twelve ovarian tumor cell lines were plated onto pre-stained N3F-derived matrices while cell-induced matrix changes were recorded and analyzed over time. All tumor cell lines but SKOV3 showed rounded morphologies when cultured within 3D matrices. Most tumor cells organized in cell clusters within the matrices with exceptions of SKOV3 and UPN251 which maintained a single cell configuration at early culturing times (data not shown). Using fluorescently pre-labeled ECM, we observed that cells with the tendency of forming clusters or agglomerates could greatly remodel the ECM compared to cells that remained as single cells (see bottom panels in Figure 1A–E for qualitative images).

Among the cells forming clusters including OVCAR5 and OVCAR10 (Figure 1A & B), the marked difference in their effects on the pre-labeled ECM allowed to categorize a majority of them into two groups. One group of cells, represented by OVCAR5, appeared to degrade the pre-labeled ECM, showing a diminished intensity of the ECM fibers immediately underneath the cell clusters (compare bottom panels of Figure 1A with 1F). The other group, represented by OVCAR10, appeared to reorganize the pre-labeled ECM as if the cells caused ‘pulling’ of ECM fibers towards areas rich in cell density, consequently exhibiting stronger fluorescent intensities in the immediate vicinity of the cell clusters (compare bottom panels of Figure 1B with 1F). These two cell groups represented by OVCAR5 and OVCAR10 can also be classified by their capability to form cell-cell interactions – the more tightly connected cells were grouped together with OVCAR5. Cells exhibiting OVCAR5-like phenotype included OVCAR2, OVCAR3, OVCAR4, PEO1, and PEO4 (Figure 1C). In contrast, A2780, and its platinum resistant sub-clones, CP70 and C30 cells [32] presented less tight clustering and matrix modification phenotypes similar to the ones observed in OVCAR10 (Figure 1E). A2780 cells also showed a degree of heterogeneity in their modifying effects on the matrices and thereby exhibited a mixed phenotype. SKOV3 and UPN251 cells (which did not form clusters) more closely resembled OVCAR5 as compared to OVCAR10 in their ability to modify ECMs (Figure 1D).

### Protease and adhesion molecule profiles differ in the two distinct matrix-modifying groups

To test if epithelial/mesenchymal and protease expression patterns could predict the type of matrix modification behaviors imparted by the various cells, we decided to assess the expression of epithelial and mesenchymal protein markers and the presence of various proteases in the panel of ovarian tumor cell lines grown under 2D conditions using Western blot analyses. We also included immortalized, non-tumorigenic human ovarian epithelium cells, HIO-80 and HIO-114 [33], [34], and human primary normal ovarian fibroblast, HFNO402 and HFNO502, as ovarian epithelial and mesenchymal cell controls.

**Figure 2:**
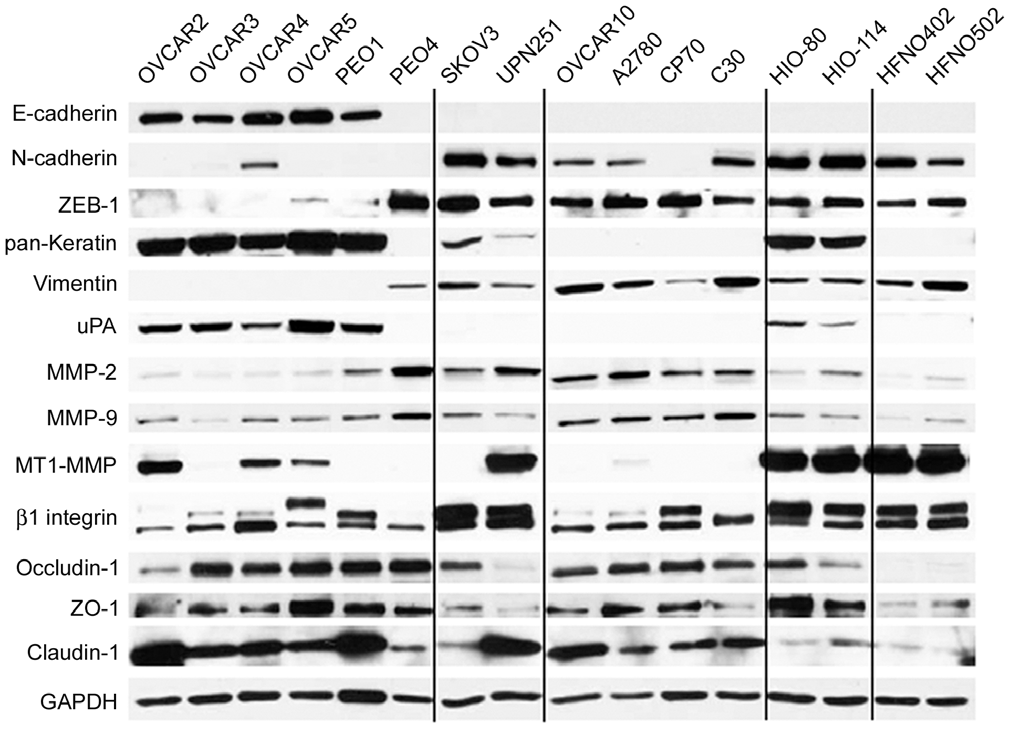
Expression of epithelial and mesenchymal markers and proteases in a panel of ovarian tumor cells. Ovarian tumor cell lysates isolated from cells grown in 2D were subjected to SDS-PAGE and immunoblotting. Cells were grouped according to their ECM remodeling capabilities shown in Figure 1. Human immortalized ovarian epithelium (HIO) and human fibroblasts derived from normal ovaries (HFNO) were used as epithelial and mesenchymal cell controls. Bands corresponding to pro-forms of uPA (55 kDa), MMP-2 (68 kDa), and MMP-9 (90 kDa) and active forms of MT1-MMP (55 kDa) were detected. Levels of glyceraldehyde 3-phosphate dehydrogenase (GAPDH) expression were used as protein loading controls.

As seen in Figure 2, the majority of the cells originally grouped with OVCAR5, with the exception of PEO4, expressed high levels of E-cadherin and keratins (an anti-pan-keratin antibody was used). In addition, these cells contained low or undetectable levels of mesenchymal markers, such as N-cadherin and vimentin, as well as ZEB-1, a transcriptional repressor of E-cadherin (Figure 2). Interestingly, PEO4 cells showed an expression pattern that is more representative of mesenchymal cells, similar to expression patterns seen in OVCAR10 cells. Indeed, cells grouped with OVCAR10 expressed vimentin and ZEB-1 and commonly lacked expression of both E-cadherin and pan-keratin (Figure 2). N-cadherin expression levels were inconsistent among cells grouped with OVCAR10, showing little or no expression in CP70, higher in C30, and intermediate in OVCAR10 and A2780 (Figure 2). As compared to cells forming clusters, SKOV3 and UPN251 cells presented with a mixed epithelial/mesenchymal phenotypes, similar patterns to the immortalized non-tumorigenic ovarian epithelial cells, HIO-80 and HIO-114. They expressed both keratin and vimentin but lacked the expression of E-cadherin. As expected, normal human ovarian epithelial cells (HIO's-) and fibroblasts (HFNO's-) presented patterns known to be associated with mesenchymal phenotypes while normal ovarian epithelial cells express keratins. Also, all cells but the normal ovarian fibroblasts expressed cell-cell interaction markers such as occludin-1, zona occluden-1 (ZO-1), and claudin-1 (Figure 2).

Additional differences between the two groups of cells were observed when patterns of protease expressions were assessed. MMP-2 and MMP-9 (pro-forms) were expressed at higher levels in cells that presented the OVCAR10 phenotype with the exception of PEO4 (Figure 2). Conversely, expression of the pro-form of urokinase-type plasminogen activator (uPA) was elevated in OVCAR5-like cells again with the exception of PEO4 (Figure 2). Active forms of membrane type 1 metalloprotease (MT1-MMP) expression levels varied but were more frequently observed in OVCAR5-like cells. β_1_-integrin expression patterns did not clearly delineate the two primary groups forming cell clusters but the expression was higher in SKOV3 and UPN251 that grew as single cells. The expression patterns of epithelial/mesenchymal protein markers were similar as observed in 2D when OVCAR5 and OVCA10 cells were cultured in 3D conditions using either N3F- or TAF-derived matrices (Figure S1).

### Localization patterns of adhesion molecules and cytoskeleton are cell type-dependent

**Figure 3:**
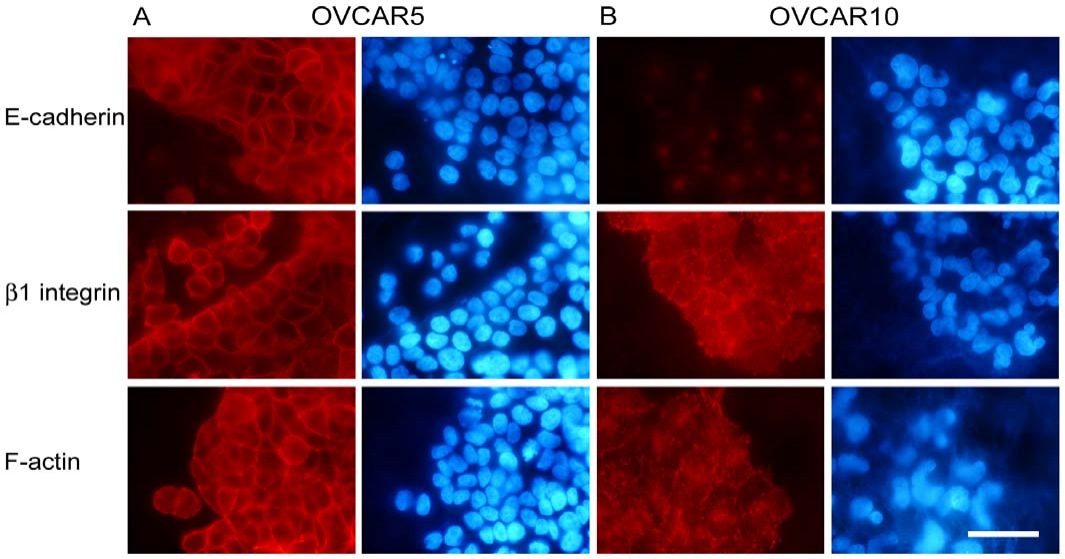
Expression patterns of E-cadherin, β_1_-integrin, and F-actin in OVCAR5 *vs.* OVCAR10 cells. OVCAR5 (A) and OVCAR10 (B) cells were plated onto N3F-derived matrices and stained for E-cadherin (top panel), β_1_-integrin (middle panel), and F-actin (bottom panel). Nuclei (blue) are shown to the right side of each panel. Bar represents 50 µm.

We next evaluated the localization patterns of E-cadherin, β_1_-integrin, and F-actin in cells cultured within N3F-derived ECMs using indirect immunofluorescence. OVCAR5 cells (which seemed to have a tight cluster phenotype) presented clear membranous E-cadherin and β_1_-integrin localizations at cell-cell contacts as well as cortical actin patterns, all suggestive of classic epithelial phenotypes that contain clear cell-cell adhesion structures (Figure 3A). In contrast, OVCAR10 cells presented no detectable E-cadherin labeling at their cell membranes while β_1_-integrin and F-actin expressions were more representative of mesenchymal-like phenotypes; presenting patched or punctuated patterns somewhat reminiscent of cell-matrix adhesion structures (Figure 3B). These differential expression patterns of adhesion molecules and cytoskeletal proteins further supported our beliefs that the presence of epithelial- or mesenchymal-like phenotypes adapted by the two types of ovarian tumor cells could contribute to cell-type dependent penetration/invasion strategies.

### Remodeling phenotypes of the ECM, indicative of penetration/invasion strategy, are cell-type specific

**Figure 4:**
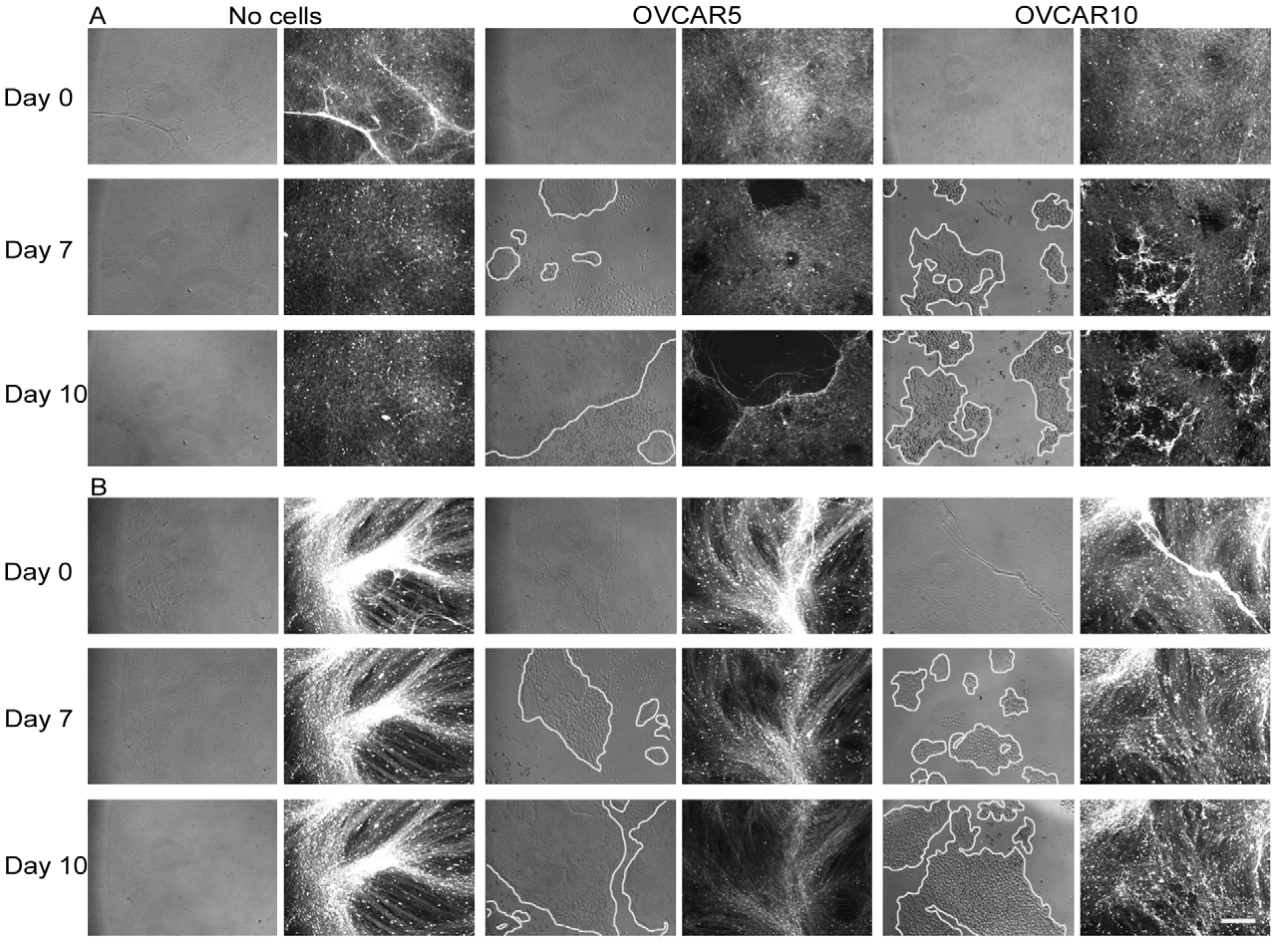
Time-lapse imaging of ECM remodeling induced by OVCAR5 or OVCAR10 cells. Pre-stained N3F- (A) or TAF-derived matrices (B) were maintained without cells or plated with OVCAR5 or OVCAR10 cells. Randomly selected locations were consistently tracked over time. Images shown were acquired on days 0 (top panel), 7 (middle panel), and 10 (bottom panel). Phase contrast (cells, left panel) and fluorescence (pre-labeled ECM, right panel) images are shown. Bar represents 200 µm.

Using time-lapse microscopy, we acquired images of pre-labeled N3F-derived ECMs before and after plating OVCAR5 and OVCAR10 cells. Matrices maintained in the absence of cells did not change over time (Figure 4A). However, in the presence of OVCAR5 cells, matrices were degraded over time and the pre-labeled material became almost undetectable following 10 days of culture (Figure 4A). In comparison, OVCAR10 cells appeared to have accumulated matrix fibers towards their proximity over time, resulting in areas which have increased fluorescent intensities (Figure 4A). Next, we conducted a similar experiment using TAF pre-labeled ECMs. The phenotypes observed using these matrices closely resembled the ones observed with N3F-derived matrices (Figure 4B).

**Figure 5:**
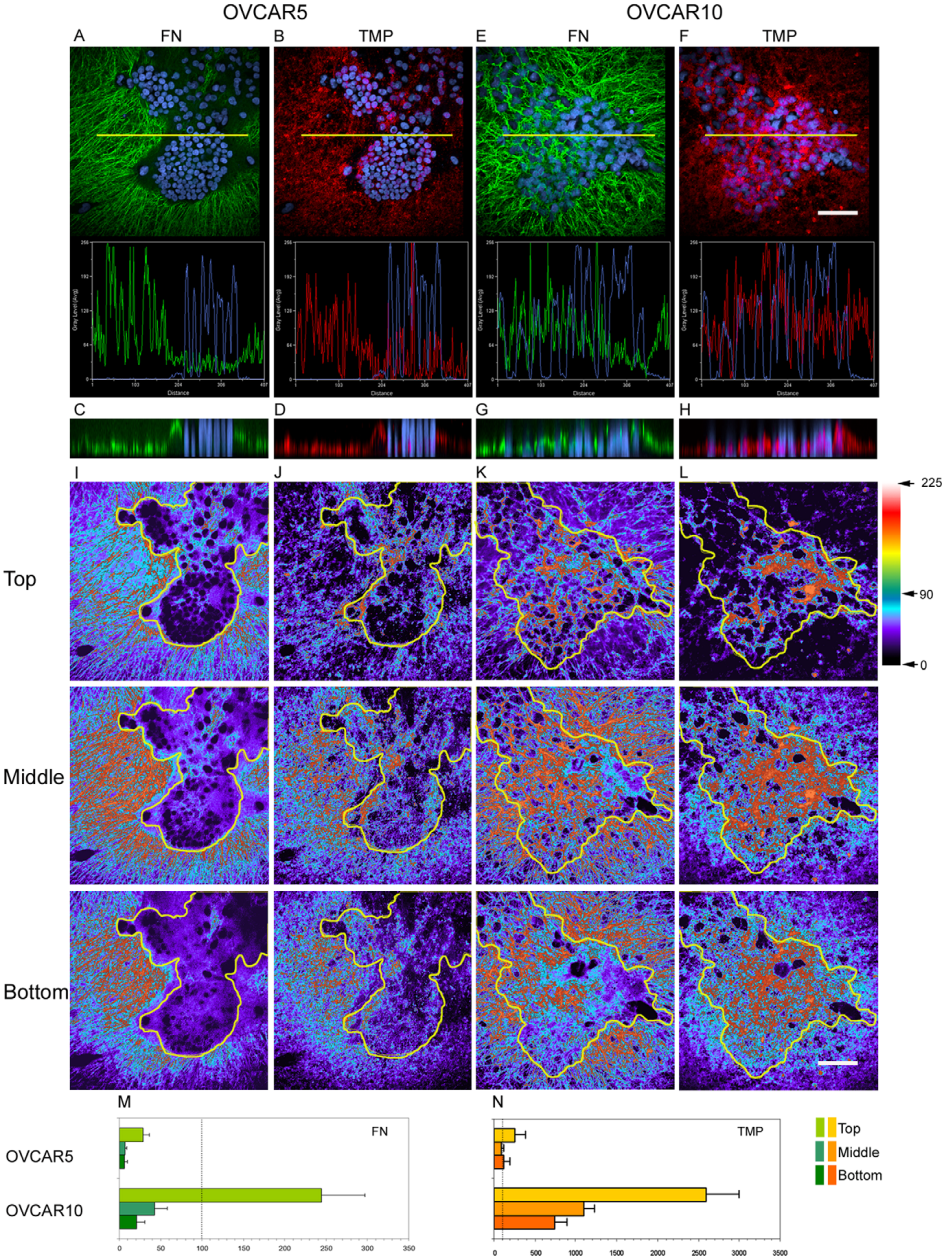
OVCAR5 or OVCAR10 cell-induced modifications in total matrix protein (TMP) and fibronectin. OVCAR5 (A–D) or OVCAR10 (E–H) cells were cultured within pre-stained N3F-derived matrices (red) for a period of 7 days and then fibronectin (green) and nuclei (blue) were detected by indirect immunofluorescence (green) and DAPI staining (blue), respectively. Images were acquired using a multiple focal plane image acquisition function to scan all files in the Z axes for each field. An XY view of 3D reconstituted maximum projections of fibronectin (A & E) and TMP (B & F) are shown. The yellow line depicts the area where the stacked images were analyzed to produce the two channel histograms shown and reconstituted to portray a maximum projection of the stack using an XZ 10 µm thick reconstitution view (C, D, G, & H). In addition, stacks were partially reconstituted into maximum XY projections using only the files corresponding to the top, middle, and bottom layers of the 3D cultures (see Materials and Methods for details). The resulting projections were pseudo-colored to match fluorescent intensity levels (I–L). Using a scale that comprises 225 levels of intensity, red/white colors depict higher intensity levels while blue/black colors depict lower intensities of fibronectin (I & K) and TMP (J & L) which were obtained in cultures containing OVCAR5 (I & J) or OVCAR10 (K & L) cells. Intensity changes induced by OVCAR5 (M) and OVCAR10 (N) cells were estimated by calculating the ratio of area corresponding to intensities that ranged between 90 and 225 (high intensity) to areas with intensities below 90 (low intensity) in both cell-containing and adjacent cell-absent areas in the same field. Cell-induced intensity change was expressed as percent difference of the ratio in cell-containing area relative to cell-absent area (100%, dotted line). Note, matrix degradation in OVCAR5 containing areas, versus accumulation of matrix by OVCAR10 cells as compared to adjacent cell-devoid area. Bar represents 100 µm.

Since OVCAR5 and OVCAR10 cells induced distinct changes on the pre-labeled ECM, we next examined whether these changes were imparted upon ECM protein in general (total matrix protein, TMP) or specifically to fibronectin. Fibronectin is one of the main components of matrices derived from fibroblasts and known for its importance in regulating matrix dynamics and collagen deposition [35], [36], [37]. Cells were plated onto TMP pre-labeled N3F-derived ECMs. Following 7 days of culture, matrices were re-stained selectively for fibronectin using a polyclonal antibody and nuclei were identified using 4′,6-diamidino-2-phenylindole (DAPI). Fluorescent intensities obtained from labeled TMP or fibronectin were compared while assessing cell-containing and adjacent (i.e., cell-devoid) areas in the same field using reconstituted projection images of the 3D cultures. The intensity of fibronectin and to a lesser degree, of pre-labeled TMP was considerably lower beneath OVCAR5 cell clusters compared to the adjacent cell-devoid areas (Figure 5A & B). Matrix thickness was greatly reduced in an OVCAR5-dependent manner (compare areas containing nuclei (blue) and areas with no nuclei in Figure 5C & D), indicating a possible degradation and/or displacement of ECMs by these cells.

Conversely, the fluorescent intensity corresponding to fibronectin and TMP did not seem to be greatly affected by OVCAR10 cells although for fibronectin there were indications of decrease in fluorescent intensity in some areas containing nuclei (Figure 5E & F). Accordingly, OVCAR10 cells did not seem to have caused appreciable change in the thickness of matrices (compare signals derived from matrix (green or red) between areas containing nuclei (blue) and areas with no nuclei in Figure 5G & H). Interestingly, matrix accumulation was evident on top of OVCAR10 cells, but not OVCAR5, (e.g., green and red traces on top of the nuclei (blue) in Figure 5G & H), suggesting the use of different strategies by the two cell groups to invade/penetrate through the ECM.

When reconstituted projection images of the 3D cultures were produced to represent the topographical top, middle, and bottom layers as shown in Figure 5I–L, the biggest difference induced by the two cell types to the matrices was observed again in the ‘top’ layers of the matrix. For example, no matrix was detected in areas containing OVCAR5 cells (Figure 5I & J) while intense signals were detected for OVCAR10 cells (Figure 5K & L, see patches of red). This cell type-dependent difference in the ‘top’ of matrices was observed both in fibronectin and TMP. When levels of fluorescent intensities obtained from TMP or fibronectin staining were quantified, results revealed that cell-containing areas retained 28% *vs.* 244% of fibronectin intensities (Figure 5M) and 258% *vs.* 2,596% of TMP intensities (Figure 5N) for OVCAR5 and OVCAR10 cells, respectively, relative to the levels detected from cell-devoid areas at the top layer. Therefore, OVCAR10 cell-containing areas presented approximately 10-fold higher intensity levels than OVCAR5 cell-containing areas relative to their cell-voided areas.

### OVCAR5- and OVCAR10-induced ECM-remodeling is dependent on the activities of proteases or ROCK, respectively

To investigate whether the cells utilize strategies that depend on ECM-degrading enzymes, integrin and/or Rho activities, we decided to assess the topographical patterns of N3F-derived ECMs modified by OVCAR5 and OVCAR10 in the presence or absence of β_1_-integrin, ROCK and different protease inhibitors. We estimated cell-induced changes of TMP and fibronectin at the three matrix locations, top, middle, and bottom under the various inhibitory conditions using confocal microscopy and image analysis as described above.

**Figure 6:**
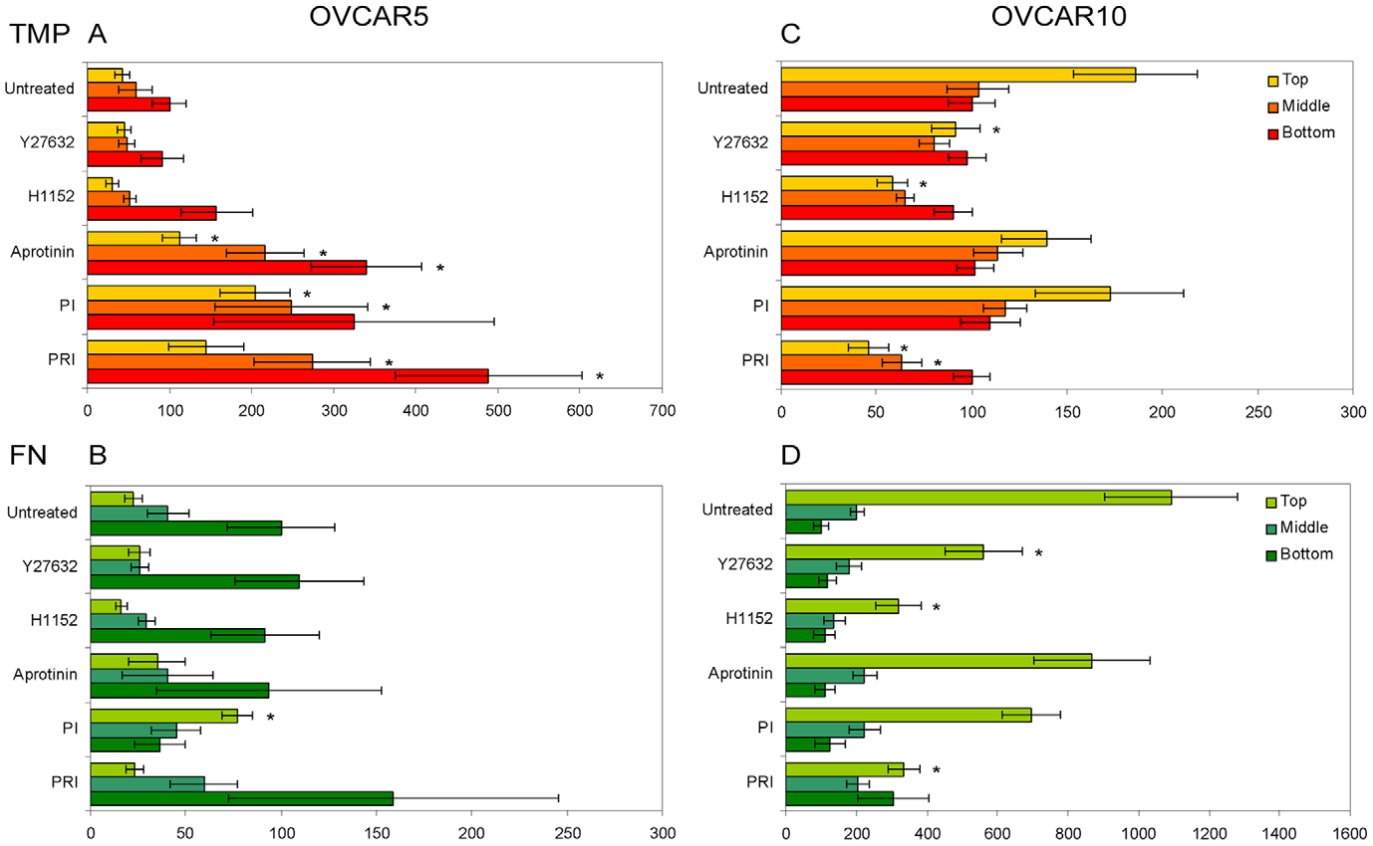
Effect of protease and ROCK inhibitors on total matrix protein (TMP) and fibronectin changes induced by OVCAR5 or OVCAR10 cells. OVCAR5 (A & B) and OVCAR10 (C & D) cells were plated onto pre-labeled N3F-derived matrices and stained for fibronectin and nuclei following 7 days of culturing under various inhibitor conditions. Images of TMP, fibronectin and nuclei staining were acquired using confocal microscopy and analyzed as described in **Figure 5**. Confocal images were reconstituted to represent the top, middle and bottom layers of the 3D cultures. Fluorescent intensity derived from TMP (A & C) and fibronectin (B & D) staining was measured. Cell-induced intensity change was estimated by calculating the ratio of area corresponding to intensities that ranged between 90 and 225 (high intensity) to areas with intensities below 90 (low intensity) in both cell-containing and adjacent cell-absent areas in the same field. Data (mean ± SE, n = 5∼10) were presented relative to intensity change at the bottom of 3D culture (100) in the absence of inhibitors (untreated control). Asterisks (*) represent significant differences (p≤0.01) compared to untreated controls using ANOVA for the top, middle, and bottom of matrices. Concentrations of inhibitors used were selected to avoid measurable inhibition of cell proliferation. PI; a protease inhibitor cocktail of individual protease inhibitors containing aprotinin (7.5 µM), leupeptin (20 µM), and GM6001 (25 µM), PRI; a mixture of PI and H1152 (0.1 µM). Refer to Table S1, Table S2, Table S3, and Table S4 for entire inhibitors tested and multiple comparisons among groups treated with different inhibitors.

OVCAR5-mediated TMP degradation (reduction of fluorescent intensity) was significantly suppressed upon treatment with aprotinin (serine protease inhibitor), a protease inhibitor cocktail (PI), and a mixture of PI and ROCK inhibitor, H1152, (PRI) as compared to untreated cells (Figure 6A and Table S1). In contrast, GM6001 (a broad spectrum MMP inhibitor) had negligible effects on TMP intensity changes while treatment of amiloride (a specific inhibitor of uPA) seemed to activate or induce additional degradation (Table S1). Therefore, we concluded that the suppression of TMP degradation by OVCAR5 cells depended on the type of protease used by the cells. Effects of protease inhibitors also appeared to be affected by ECM components. Different from TMP, fibronectin intensity was not significantly affected by the presence of protease inhibitors in OVCAR5 cell-mediated invasion - only PI (Figure 6B) and β_1_-integrin antibody (Table S2) were able to significantly reduce fibronectin degradation and their effects were limited only to the ‘top’ layer. In comparison, TMP and fibronectin were similarly affected by inhibitors used in the presence of OVCAR10 cells. OVCAR10-induced intensity changes on the ECM were largely suppressed by ROCK inhibition using H1152 or Y27643 and by PRI (Figure 6C & D and Tables S3 & 4). However, none of protease inhibitors tested effectively inhibited OVCAR10-induced intensity changes (Figure 6C & D and Tables S3 & 4).

Furthermore, there was a strong correlation between the TMP confocal microscopy data and results obtained using epifluorescence microscopy. The penetration/invasion of OVCAR5 cells within N3F-derived ECMs could be inhibited in the presence of aprotinin, PI, and PRI, while leupeptin, GM6001, or amiloride did not effectively suppress the OVCAR5-induced ECM degradation; i.e., the resultant ECMs appeared to be impossible to differentiate from the ones using untreated controls (Figure S2A). Conversely, none of the protease inhibitors tested appeared to effectively inhibit OVCAR10-induced ECM changes (Figure S2B). ROCK inhibitors (Y27632 and H1152) did not show any effects on OVCAR5-induced ECM modifications (Figure S2A), while these inhibitors clearly reduced ECM changes induced by OVCAR10 cells (Figure S2B). Finally, the functional blockage of β_1_-integrin did not appear to affect either OVCAR5- or OVCAR10-induced changes to the underlying ECM (Figure S2A & B). In all cases, controls using vehicle (DMSO) or non-specific IgG antibodies did not affect the cell-induced matrix modification (data not shown).

The inhibitory effect of protease and ROCK inhibitors was next evaluated in an expanded panel of ovarian tumor cells. Epifluorescence images of pre-labeled N3F-derived matrices indicated that the ECM modification induced by OVCAR3 and OVCAR4 cells were largely inhibited by aprotinin but not H1152 (Figure S3A). Conversely, ECM accumulation induced by CP70 and C30 was inhibited by H1152 whereas aprotinin did not affect (Figure S3B). Therefore, cell-induced ECM degradation was inhibited by aprotinin while ECM contraction was suppressed by H1152.

### Cells that degrade ECMs secrete higher caseinolytic enzymes than cells that tend to contract ECMs

**Figure 7:**
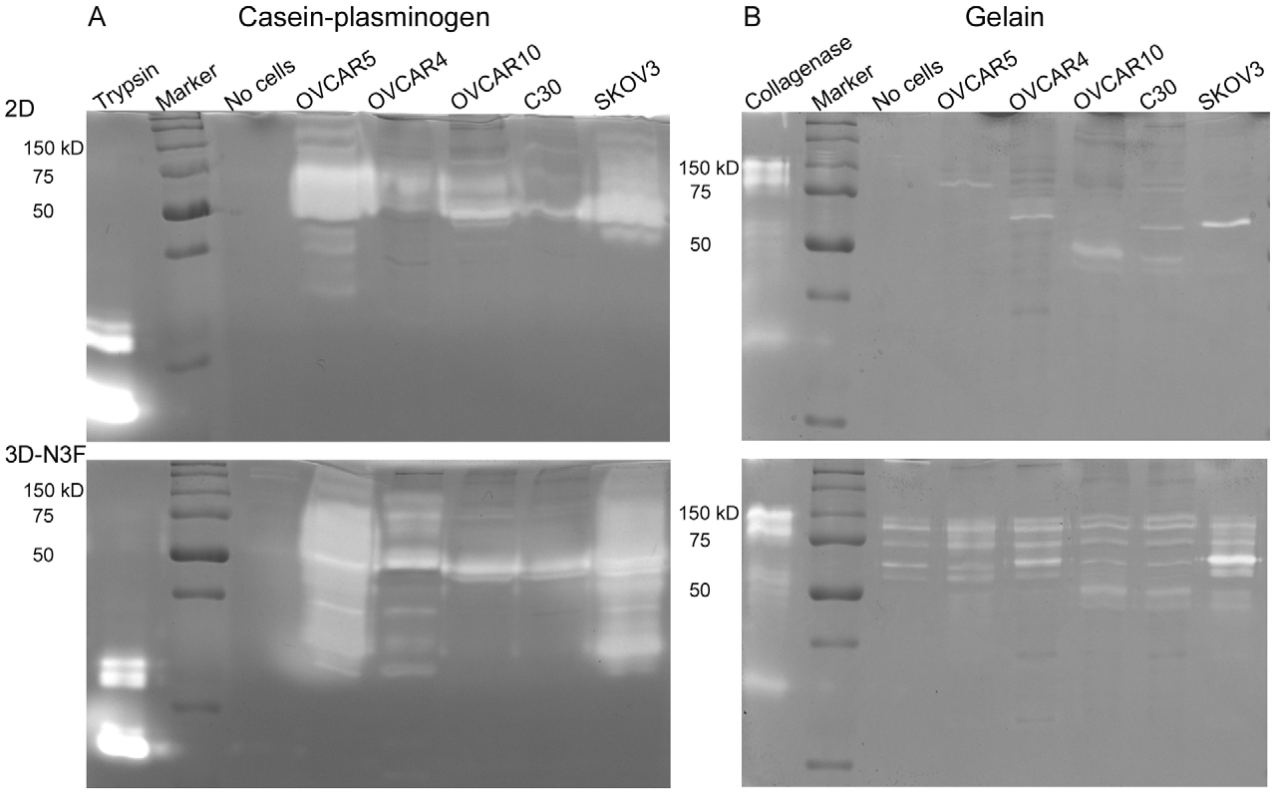
Differential activity of caseinolytic and gelatinolytic enzymes secreted by cells grouped according to their ECM remodeling phenotypes. Representative cells in each ECM remodeling category were cultured in 2D (top panels) and 3D (N3F-derived matrices, bottom panels) conditions. Conditioned media were subjected to SDS-PAGE using gels copolymerized with casein and plasminogen (A) or gelatin (B). Trypsin and collagenase were used as positive controls of caseinolytic and gelatinolytic activity, respectively. Note that culturing OVCAR5, OVCAR4, and SKOV3 cells in 3D conditions increased caseinolytic activity, especially enzyme species in the molecular weight range of 30∼50 kDa.

ROCK and protease activities were assessed in cells grown in either 2D or 3D conditions and compared the result with the sensitivity to protease and ROCK inhibitors. Zymography was used to detect protease activity of conditioned media derived from cells representing the ECM modifying phenotypes. Interestingly, OVCAR5, OVCAR4, and SKOV3 cells that degrade the ECM as either group or single cells secreted higher levels of caseinolytic proteases both in 2D and 3D (N3F-derived ECMs) cultures as compared to OVCAR10 and C30 cells that cause ECM contraction (Figure 7A). This difference was even more apparent in 3D conditions where caseinolytic enzyme species lower than size of 50 kDa appeared to be highly activated (Figure 7A). Similarly, OVCAR5, OVCAR4, and SKOV3 cells appeared to secrete more gelatinolytic proteases in 3D compared to 2D conditions (Figure 7B). Noticeably, N3F-derived matrices contain a background level of gelatinolytic enzymes (Figure 7B). Overall, protease activities derived from OVCAR10 and C30 cells were minimal and did not appear to be further induced under the 3D condition (Figure 7A & B). These results correlated well with the observation that aprotinin inhibit ECM modification by OVCAR5- but not OVCAR10-like cells (Figure S3A & B). Also, the result implicates that MMP inhibitors may not be effective in inhibition of ECM remodeling by either groups of cell that secret low levels of gelatinolytic enzymes (Figure 7B). Cells that degrade ECMs contained multiple caseinolytic enzyme species (Figure 7A) not inhibited by a specific uPA inhibitor (Figure S4), explaining the lack of inhibition of ECM degradation by amiloride (Figure S2A and Tables S1 & 2).

Finally, we evaluated if cellular ROCK activity is associated with cell type-dependent inhibitory effect of the ROCK inhibitors. ROCK activities were similar among cells cultured in either 2D or 3D (N3F-derived matrices) conditions with the exception of OVCAR2, SKOV3, and UPN251 cells (Figure S5). Therefore, cellular levels of ROCK activity alone did not appear to correlate well with cell type-dependent ECM modifying phenotypes.

## Discussion

Ovarian cancer is a highly aggressive disease, making inhibition of adjacent organ penetration/invasion, which facilitates the dissemination of this cancer, an important therapeutic goal. Mesenchymal cell migration accompanied by EMT has been suggested to be the major invasion mechanism of ovarian cancer cells [9]. However, recent studies, using 3D culture models, revealed that cancer cells can use additional strategies to invade through tissues [17], [26], [38]. As such, cells have been shown to be capable of invading without disrupting cell-cell interactions (i.e., collective cell migration) or to penetrate the ECM without major protease or integrin dependencies (i.e., amoeboid cell migration) [23]. Since ovarian cancer cells often acquire a peculiar phenotype which includes up-regulation of E-cadherin expression in contrast to its absence in normal OSE [4], we hypothesized that these cells may penetrate/invade through ECMs using alternative strategies to the well-studied mesenchymal cell migration. Therefore, the matrix remodeling capabilities of a panel of ovarian tumor cell lines was assessed using fibroblast-derived matrices, to mimic ECMs of *in vivo* environments [30].

Different ECM topographic changes were induced by various tumor cells. A group of cells, represented by OVCAR5, appeared to have degraded the ECM. They also seemed to maintain their cell-cell contact while they invaded through ECMs (Figure 1A & C) and clearly expressed cell-cell adhesion markers (Figures 2 & 3A). The other primary group, represented by OVCAR10, appeared to modify the ECM by a massive rearrangement (e.g., contraction or accumulation) of the ECM (Figure 1B & E). These cells presented a more loose agglomeration (Figures 2 & 3B). A potential third group, i.e., SKOV3 and UPN251, appeared to behave in a single cell manner with less clear impairment or remodeling upon ECMs (Figure 1D). Some of previous studies suggested that cell morphology implicates cell invasion strategies [17], [23], [24]. However, in our study, most of tumor cell lines presented an epithelial phenotype in 2D cultures and showed rounded morphology within the matrices suggesting that cell morphology itself may not be sufficient to predict the type of ECM remodeling and subsequently, the penetration/invasion strategy. We conducted similar studies using human ovarian fibroblast-derived matrices. Although it was less apparent, tumor cells (e.g., OVCAR5 and OVCAR10) presented similar ECM modifying effect as observed in N3F- or TAF-derived matrices (data not shown). Human fibroblasts produced matrices visibly very different from N3F-derived matrices when they were fluorescently stained, i.e., substantially thinner and less uniform, making it more difficult to assess cell behavior. In addition, differences in architecture of the ECM as well as protein composition might explain differences between the ECM modifying behavior on N3F- and human fibroblast-derived matrices. For instance, cancer cells may have a limited ability to migrate through a rigid pore [39], therefore, they are likely to rely on ECM-degrading enzymes to invade through rigid ECMs. Also, cells may prefer invading through N3F-derived matrices which lack type I collagen, which is more resistant to enzymatic degradation than other ECM components (e.g., fibronectin), compared to human fibroblast-derived matrices rich in type I collagen (data not shown).

We conducted most of our studies using cells that represented the two distinct phenotypes of collective agglomerates within ECMs, OVCAR5 and OVCAR10. These cells induced strikingly different topographic changes to the ECM while making their way through these substrates (Figures 4 & 5). We believe that the matrix remodeling phenotype could be used to assess penetration/invasion strategies. For example, based on matrix remodeling phenotypes, OVCAR5 cells fit into a ‘collective cell migration’ category where cells migrate as groups and use ECM-degrading proteases for their invasion [17]. In fact, this phenotype was also observed when OVCAR5 cells were evaluated as spheroids invading through a monolayer of mesothelial cells [40], thus confirming the relevance of our approach. Collective cell migration has also been demonstrated in colon cancer cells by Nabeshima and colleagues [22], [41], [42], [43]. Their study showed that the expression of ECM-degrading proteases, such as MT1-MMP and MMP-2 at the leading edge of the cell aggregates are critical for cohort migration, a type of collective cell movement [22]. More recently, it was reported that podoplanin, a plasma membrane glycoprotein, induces tumor cell migration and invasion without disrupting E-cadherin-mediated cell-cell junction both *in vitro* and in a transgenic model of carcinogenesis *in vivo* [12]. In contrast, ECM rearrangement mediated by OVCAR10 cells, similar to patterns of collagen contraction induced by mesenchymal cells [44], [45] did not seem to either rely on proteases or present tight cell-cell interaction as seen in OVCAR5. In addition, protein localization patterns of E-cadherin, β_1_-integrin, and F-actin of the two cell lines suggested that OVCAR5 and OVCAR10 cells indeed present epithelial *vs.* mesenchymal phenotypes, respectively (Figure 3A & B). β_1_-integrin expression at cell-cell contact in OVCAR5 cells (Figure 3A) also indicated very tight cell-cell interactions [46].

Evaluation of protein expression profiles further confirmed epithelial and mesenchymal characteristics of two groups of cells represented by OVCAR5 and OVCAR10. All cells grouped together with OVCAR5, except for PEO4, showed a distinct epithelial phenotype, i.e., expressed E-cadherin and pan-keratin with little or no expression of vimentin, N-cadherin, and ZEB-1, an E-cadherin repressor (Figure 2). Conversely, cells grouped with OVCAR10 cells expressed a battery of proteins reminiscent to those expressed in mesenchymal cells, i.e., expressed vimentin and ZEB-1 but lacked pan-keratin and E-cadherin. Nevertheless, these cells also presented some epithelial markers such as claudin-1, ZO-1, and occludin-1, which are typically localized at tight junctions and are often lost during EMT [47], suggesting that this cell may be in partial EMT [48], [49]. We also found differences in protease profiles among the cells with the abovementioned epithelial and partial EMT phenotypes (Figure 2). For example, uPA (pro-form) was only expressed in cells that presented the epithelial properties. Most of the ovarian tumor cells expressed MMP-2 and MMP-9 (pro-forms) weakly as previously observed [50]; however, the tumor cells with partial EMT phenotypes had higher levels. Therefore, the two groups could be divided by the differential protease profile as well. Nevertheless, inhibitors of these proteases did not seem to have an effect in reversing any of the two phenotypes studied probably due to differences between cellular expression levels and secreted protease activity as discussed below. In contrast, β_1_-integrin expression was relatively uninformative in grouping OVCAR5- and OVCAR10-like cells (Figure 2). However, we did observe that β_1_-integrin expression might be useful to discriminate cells degrading ECM as single cells (SKOV3 and UPN251) from those that induce ECM rearrangement (e.g., OVCAR10). Previous studies have reported that β_1_-integrin expression is reduced in protease-independent amoeboid cell migration compared to protease-dependent mesenchymal cell migration [24].

Overall, our studies demonstrated that protein expression profiles of adhesion molecules, cytokeratin, and proteases could predict the strategy of penetration/invasion using an *in vitro* 3D model, although, there were some exceptions. As discussed, PEO4 cells were grouped with OVCAR5 cells based on their ECM remodeling properties (Figure 1C); however, their protein profile was similar to OVCAR10 cells (Figure 2). We speculate that the ECM change induced by PEO4 cells might be smaller than other cells presenting OVCAR10-like phenotypes; thereby, they might not be grouped correctly based on detection of fluorescent signal after prolonged culture. Importantly, PEO1 and PEO4 cell lines were obtained from the same patient before and after the onset of resistance to chemotherapy [35], [51], and may be reflective of a major problem in treating ovarian cancer patients, i.e., the subsequent development of drug resistance and recurrence of cancer in spite of initial effectiveness of platinum-based chemotherapies [52]. One can speculate that platinum-based chemotherapy might result in the switching of a given invasion mechanism and evolvement of additional strategies. Nevertheless, these types of questions are beyond the scope of this study.

Based on the above observed characteristics, we anticipated that ECM modification induced by OVCAR5 cells would be blocked by protease inhibitors, while ROCK inhibitors would suppress ECM modifications imparted by OVCAR10 cells which may have an ability to generate force to contract ECMs. The contraction of actin filament is known to be primarily induced by small G-protein Rho and its downstream effector, ROCK, responsible for protease independent invasion (e.g., amoeboid) [53], [54], [55], [56]. Upon exposure to inhibitors of different proteases, the degradation of TMP by OVCAR5 cells was greatly prevented. However, the effect was protease type-dependent. Aprotinin, a serine protease inhibitor, effectively inhibited OVCAR5 cell-mediated TMP degradation (Figures 6A & S2A). In contrast, the effect of leupeptin, which inhibits both serine and cysteine proteases was minimal, as was GM6001, a broad range MMP inhibitor. These results correlated with the detection of massive caseinolytic but negligible gelatinolytic proteases secreted by OVCAR5 cells cultured within N3F-derived matrices (Figure 7A & B). We expected that amiloride, known to specifically inhibit uPA but not tPA [57], would effectively prevent OVCAR5 cells from degrading the ECM since these cell type expressed higher uPA (Figure 2). However, amiloride appeared to enhance TMP degradation (Table S1). In fact, OVCAR5 cell-conditioned media contained caseinolytic proteases not inhibited by amiloride especially at molecular weight above 50 kDa (Figure S4). Noticeably, no protease inhibitors effectively prevent fibronectin degradation by OVCAR5 cells (Figure 6B and Table S2), implying that fibronectin may be more susceptible to enzymatic degradation and substrates of many different types of proteases. These results suggest that different proteases have specificities for different ECM components and that it will be important to determine protease type secreted by both tumor and stromal cells to predict their specific ECM remodeling strategies.

ROCK inhibitors blocked OVCAR10 cell-induced invasion and ECM contraction (Figures 6C & D and S2B). However, cells with an OVCAR10-like phenotype did not appear to have higher ROCK activity as measured by an enzymatic immunoassay (Figure S5). It has been reported that invasion of cells which did not express ROCK or Rho at high levels were also inhibited by a ROCK inhibitor [58]. Therefore, other mechanisms regulating ROCK activity may be responsible for ROCK-dependent ECM modification by these cells. MMP activity has been considered to be implicated in collagen gel contraction and tissue reorganization as well [59], [60], [61]. However, the effect of GM6001 was negligible on ECM contraction by OVCAR10 cells in our study, as observed previously in Hey and ES-2 ovarian tumor cells [62]. Also, the addition of a cocktail of protease inhibitors did not seem to further enhance the inhibitory effect of the ROCK inhibitors (Figure 6C & D), and therefore, none of the protease inhibitors we tested appeared to be effective in OVCAR10-induced matrix change. This might be explained by low protease activity derived from these cells (Figure 7A & B). Importantly, aprotinin and H1152, which effectively inhibited OVCAR5- and OVCAR10-induced ECM modification, respectively, also inhibited ECM changes induced by other cells grouped together (Figure S3A & B). Therefore, two different cell types divided by epithelial and mesenchymal phenotypes contribute to different ECM phenotypes, degradation and accumulation or contraction of ECMs, respectively, and their ECM modification can be reversed by the use of specific protease and ROCK inhibitors, respectively.

In conclusion, we have shown for the first time using a physiologically relevant 3D model that ovarian tumors cells that present epithelial characteristics tend to penetrate/invade the mesenchymal ECM as clusters or groups and use proteases to degrade the matrix. On the other hand, cells that present partial EMT phenotypes may push through the ECM by mechanisms that are based on Rho-dependent ECM accumulation or contraction and are less dependent on proteolysis for their penetration/invasion. Our study also expands upon the view that many cell types including tumor cells naturally penetrate through ECMs while invading without the use of classic EMT mechanisms. Our study suggests that differential ECM modification mechanisms by various ovarian tumor cell lines may reflect heterogeneity among tumors from different patients or within a given tumor. Therefore, fully characterizing the potential invasion mechanisms may help to design therapies targeting theses ovarian cancer cell behaviors.

## Materials and Methods

### Cell cultures

NIH-3T3 fibroblast (N3Fs, originally obtained from ATCC) were pre-conditioned in media containing 10% fetal bovine serum (FBS) during serially cultivating for at least 22 passages [28], [29] before the use for 3D matrix production and maintained in Dulbecco's modified Eagle's medium supplemented with 10% FBS, 2 mM L-glutamine, 100 U/mL penicillin, and 100 µg/mL streptomycin. Primary fibroblasts [28] were isolated from mouse skin tumor induced by two stage carcinogenesis regimen and characterized as tumor-associated fibroblast (TAF) [28], [31]. These TAFs (passage 5 to 7) were maintained in the same medium as above. De-identified human ovarian tissue not required for diagnosis was obtained from the FCCC Biosample Respository following informed consent and HFNO402 and HFNO502 primary fibroblast cultures were derived as previously described [63] under a protocol approved by the FCCC institutional review board. The panel of ovarian tumor cell lines used consisted of OVCAR2, OVCAR3, OVCAR4, OVCAR5, OVCAR10, A2780, CP70, C30, PEO1, PEO4, SKOV3, and UPN251 [32], [35], [64]. All tumor cells were cultured in RPMI 1640 medium supplemented with 10% FBS, 0.3 U/mL insulin, 2 mM L-glutamine, 100 U/mL penicillin, and 100 µg/mL streptomycin. All the cells were cultured in a humidified incubator at 37°C and 5% CO_2_. All the reagents formulated for media were purchased from Mediatech, Inc. (Manassas, VA) except for insulin (Sigma-Aldrich, St. Louis, MO).

### Fibroblast-derived 3D matrix production

*In vivo*-like 3D matrices were derived from N3Fs (for the majority of studies) or TAFs as previously described [28], [29], [30], [31]. Briefly, cells were plated at the density of 2.5×10^5^ cells/mL and treated with freshly prepared 50 µg/mL ascorbic acid every 48 hours for 6 days after they reached confluence. Following the ascorbic acid treatment, fibroblasts were removed from the ECM using phosphate buffered saline (PBS) containing 0.5% (v/v) Triton® X-100 and 20 mM ammonium hydroxide (Sigma-Aldrich). Resulting cell-free 3D matrices were washed and stored in PBS supplemented with 100 U/mL penicillin and 100 µg/mL streptomycin at 4°C until used.

### Antibodies and inhibitors

Anti-E-cadherin (Clone 36), N-cadherin (32), and β_1_-integrin (18) antibodies were obtained from BD Biosciences (San Diego, CA) and used for immunofluorescence (IF) staining and immunoblotting analyses. Other antibodies used for immunoblot analysis were pan-keratin (80, Abcam Inc., Cambridge, MA), vimentin (VIM-13.2, Sigma-Aldrich), uPA (AB-2, NeoMarkers, Fremont, CA), MT1-MMP (LEM-2/15.8, Chemicon International, Temecula, CA), MMP-2 (polyclonal, Abcam Inc.), MMP-9 (GE-213, Chemicon International), ZO-1 (Z-R1, Zymed, San Francisco, CA), Claudin-1 (JAY.8, Zymed), Occludin-1 (OC-3F10, Zymed), ZEB-1 (polyclonal, Bethyl Laboratories Inc., Montgomery, TX), and GAPDH (Chemicon International). Protease inhibitors, aprotinin, leupeptin, and amiloride were obtained from Sigma-Aldrich and included in the media at concentrations of 7.5, 20, and 100 µM, respectively. A broad spectrum inhibitor of MMP, GM6001 (25 µM), and Rho Kinase inhibitors, Y27632 (25 µM), and H1152 (0.1 µM) were purchased from EMD Chemicals, Inc (Gibbstown, NJ). Monoclonal anti-β_1_ integrin (6S6, azide free) was purchased from Chemicon International and added in culture medium (0.5 µg/mL) to inhibit functional activity of β_1_-integrin. The same concentration of mouse IgG (Chemicon International) was used as a negative control. Concentration of inhibitors was determined at the levels which did not significantly affect cell viability in 2D culture using CellTiter Blue Cell Viability Assay reagent (Promega, Madison, WI) and EnVision Mutilabel Plate Reader (PerkinElmer, Waktham, MA).

### ECM penetration/invasion assays

ECMs were derived from N3Fs or TAFs in 24-well plates and stained with 1 µg/mL Alexa Fluor 555 carboxylic acid, succinimidyl ester (Invitrogen, Carlsbad, CA) overnight, at 4°C and then washed with PBS. Ovarian tumor cells (1,000 to 8,000 cells/well depending on the cell line) were plated onto pre-stained matrices and imaged every 2 days for 14 days after plating. Some matrices were maintained with media in the absence of cells and served as negative controls. Bright-field (cells) and fluorescence (matrices) images were acquired using Nikon TE300 Inverted Fluorescent Microscope (Nikon Inc., Melville, NY) equipped with image acquisition and processing software, MetaVue (Molecular Devices, Downingtown, PA). In order to track the same field over time, 9 randomly preselected fields per well were repeatedly imaged 0, 7, and 10 days after plating cells. For penetration/invasion inhibition, 24 hours after seeding cells, the media was replaced with freshly prepared media containing the inhibitors listed above or their controls (e.g., water, dimethyl sulfoxide; DMSO, and IgG). Inhibitor-containing media were replaced every 48 hours and images were acquired at 7 days of culture. All the experiments were conducted in duplicates and repeated at least three independent times.

### Indirect immunofluorescence staining

Tumor cells cultured within N3F-derived matrices were fixed in 4% paraformaldehyde, permeabilized with 100 µM digitonin, and stained for β1 integrin, E-cadherin, and F-actin (phalloidin conjugated to the tetramethyl rhodamine, Invitrogen). For fiber detection of fibronectin (Abcam Inc.), 3D cultures were first permeabilized with 4% paraformaldehyde containing 0.5% Triton® X-100 for 3 minutes and then further fixed for 20 minutes using 4% paraformaldehyde containing 5% glucose. Fixed/permeabilized samples were blocked using 5% BSA dissolved in PBS. Following blocking, cover slips were incubated with primary antibodies for 1 hour. Alexa Fluor 488 anti-rabbit IgG or Alexa Fluor 594 anti-mouse IgG (Invitrogen) were used as secondary antibodies. Immunostained cover slips were mounted with Vectashield containing DAPI (Vector Laboratories, Inc, Burlingame, CA) and visualized using Nikon Eclipse E800 Fluorescent Microscope.

### Confocal microscopy and image analysis

Images were taken using Nikon TE2000 Eclipse Inverted Microscope equipped with C1 scan head and operated by EZ-C1 software (Nikon Inc.). A minimum of five random fields per sample were selected. Each field was scanned using a multiple focal plane acquisition mode where images were taken at 0.5 µm intervals using wavelengths corresponding to 405 (blue), 488 (green), and 561 nm (red), thus detecting nuclei, fibronectin, and total matrix protein (TMP), respectively. MetaMorph (version 7.0, Molecular Devices, Downingtown, PA) was used to acquire a maximum intensity projection of a 3D image stack comprising of all focal planes obtained from each field and to analyze images acquired. Tumor cells present in the multilayer matrices were evaluated using 90 degree reconstruction images. Cell behaviors within ECMs in 3 different layers from the top to the bottom of the matrices were also measured. From the original entire 3D reconstruction image, z-planes were separately reconstituted to represent ‘top,’ ‘middle,’ and ‘bottom’ fractions of the 3D cultures as defined below. ‘Top,’ the plane on which one could start detecting at least 20% of the field with stain-positive area for fibronectin fibers and one additional plane below. ‘Bottom,’ the plane where fibronectin staining is detected in an entire field and one additional plane above. Finally, ‘middle,’ the one or two halfway planes of all the planes encompassing from ‘top’ to ‘bottom’. Reconstituted images corresponding to each of these three topographic fractions were pseudo-colored according to their labeled (immunofluorescent) TMP or fibronectin intensities, which ranged between 0 (the lowest) to 225 (the highest). Changes in TMP or fibronectin induced by cells were estimated by calculating the ratio of area (the total number of pixels) corresponding to intensities that ranged between 90 and 225 (high intensity) relative to areas with intensities below 90 (low intensity) in both cell-containing and adjacent cell-absent areas in the same field. Cell-induced intensity change was expressed as percent difference of the ratio in cell-containing area relative to cell-absent area (100%). Cell-containing areas were selected by manually circumventing around cell clumps detected by cell nuclei (DAPI) staining using original reconstituted projection image of the entire 3D culture in each field and designated as ‘cell region’.

### Immunoblot analysis

Cells cultured in 2D or under 3D (N3F-derived ECMs) conditions were lysed using radioimmunoprecipitation assay buffer (50 mM Tris HCl, pH 8.0, 5 mM EDTA, 150 mM NaCl, 1% NP40, 0.5% sodium deoxycholate, and 0.1% SDS) supplemented with protease inhibitor cocktails (Sigma-Aldrich). Cell homogenates were centrifuged at 10,000×g for 5 min at 4°C. Aliquots of supernatant were mixed with sample loading buffer (50 mM Tris buffer pH 6.8, 10% glycerol, 2% SDS, 5% β-mercaptoethanol, and 0.02% bromophenol blue) and denatured at 95°C for 5 min. Denatured proteins (35 µg/lane) were loaded onto 10% or 4–20% Novex® Tris-Glycine gels (Invitrogen). After electrophoresis, proteins were transferred onto nitrocellulose membranes and probed with the indicated antibodies. Protein detection was achieved using Western Lightning TM Plus-ECL Enhanced Chemiluminescence Substrate (Perkin Elmer, Waltham, MA). Protein concentration was measured using DC Protein Assay reagents (Bio-Rad, Hercules, CA).

### Gelatin and casein-plasminogen zymography

Cells were cultured in 2D or 3D (N3F-derived matrices) conditions until they reached ∼70% confluence and the media was replaced with serum-free media for 48 hours. Supernatants were concentrated by ultra-filtration using Amicon Ultra-15 centrifugal filter device (Millipore Corporation, Bedford, MA) at 3,000×g for 1 hour at 4°C. Concentrated samples (10 µg) were subjected to non-reducing SDS-PAGE as described in the previous study [50] using gels co-polymerized with 0.1% gelatin or 0.1% casein and 10 µg/mL plasminogen (Sigma-Aldrich).

### Statistical analyses

One-way analysis of variance (ANOVA) was used to compare effect of different inhibitors on fibronectin or TMP intensity change induced by cells using SAS software (version 9.2, SAS Institute Inc, Cary, NC). Benjamin and Hochberg method was used to control for the multiple comparisons and the false discovery rate, which was controlled at 0.05. P-values smaller or equal to 0.01 were considered significant. Data were converted to log scale for analyses due to differences in the variance among groups compared together.

## Supporting Information

Figure S1 Expression of epithelial and mesenchymal markers in OVCAR5 and OVCAR10 cells grown in 2D and 3D (N3F- and TAF-derived matrices) conditions. Note that matrices (N3F and TAF-derived) did not contribute to appreciable amount of any proteins tested. For cell lysates obtained from 3D cultures, matrices maintained without cells were used as controls to subtract proteins derived from matrices.
(TIF)

Figure S2 Effect of various inhibitors on matrix remodeling induced by OVCAR5 and OVCAR10 cells. OVCAR5 (A) and OVCAR10 (B) cells were plated onto pre-labeled N3F-derived matrices and cultured under various inhibitory conditions. Phase contrast (cells, top panel) and fluorescence (matrices, bottom panel) images were acquired at 7 days of culture. Bar represents 200 µm. Concentrations of inhibitors used were selected to avoid noticeable inhibition of cell proliferation. PI; a protease inhibitor cocktail of individual protease inhibitors containing aprotinin (7.5 µM), leupeptin (20 µM), and GM6001 (25 µM), PRI; a mixture of PI and H1152 (0.1 µM).
(TIF)

Figure S3 Effect of aprotinin and H1152 on matrix remodeling induced by cells with epithelial and partial EMT phenotypes. OVCAR5-like cells, e.g., OVCAR5, OVCAR3, and OVCAR4 (A), and OVCAR10-like cells, e.g., OVCAR10, CP70, and C30 (B), were plated on pre-labeled N3F-derived matrices and cultured in the absence or presence of aprotinin (7.5 µM) and H1152 (0.1 µM). Phase contrast (cells, top panel) and fluorescence (matrices, bottom panel) images were acquired at 7 days of culture. Bar represents 200 µm. Note that aprotinin effectively inhibited ECM modification induced by OVCAR5, OVCAR3, and OVCAR4 cells which degrade ECMs in contrast to suppression of ECM contraction induced by OVCAR10, CP70, and C30 cells by H1152.
(TIF)

Figure S4 Amiloride on caseinolytic activity derived from OVCAR5 and OVCAR10 cells. Conditioned media derived from 3D (N3F-derived matrices) cultures of OVCAR5 and OVCAR10 cells were subjected to SDS-PAGE using gels copolymerized with casein and plasminogen. Casein gels were incubated with developing buffer for overnight at 37°C in the absence and presence of an uPA inhibitor, amiloride. Note that caseinolytic activity was retained even after the treatment of 1 mM amiloride.
(TIF)

Figure S5 ROCK activity in a panel of ovarian tumor cells. Ovarian tumor cell lysates isolated from cells grown in 2D or 3D (N3F-derived matrices) were subjected to an enzymatic immunoassay using ROCK Activity Assay Kit (see File S1 for details). Cells were grouped according to their ECM remodeling capabilities as shown in Figure 1. ROCK activity was expressed as units (in pg) of purified active ROCKII.
(TIF)

File S1 Supplemental Methods.
(DOC)

Table S1 Intensity change of total matrix protein induced by OVCAR5 cells in the presence and absence of various inhibitors measured at the top, middle, and bottom parts of 3D culture.
(DOC)

Table S2 Intensity change of fibronectin induced by OVCAR5 cells in the presence and absence of various inhibitors measured at the top, middle, and bottom parts of 3D culture.
(DOC)

Table S3 Intensity change of total matrix protein by OVCAR10 cells in the presence and absence of various inhibitors measured at the top, middle, and bottom parts of 3D culture.
(DOC)

Table S4 Intensity change of fibronectin induced by OVCAR10 cells in the presence and absence of various inhibitors measured at the top, middle, and bottom parts of 3D culture.
(DOC)
