# Supplementary figures and images for: Differential Expressions of Adhesive Molecules and Proteases Define Mechanisms of Ovarian Tumor Cell Matrix Penetration/Invasion
Source: PLoS One. 2011 Apr 19;6(4):e18872. doi: 10.1371/journal.pone.0018872 (PMC3079735; doi:10.1371/journal.pone.0018872)

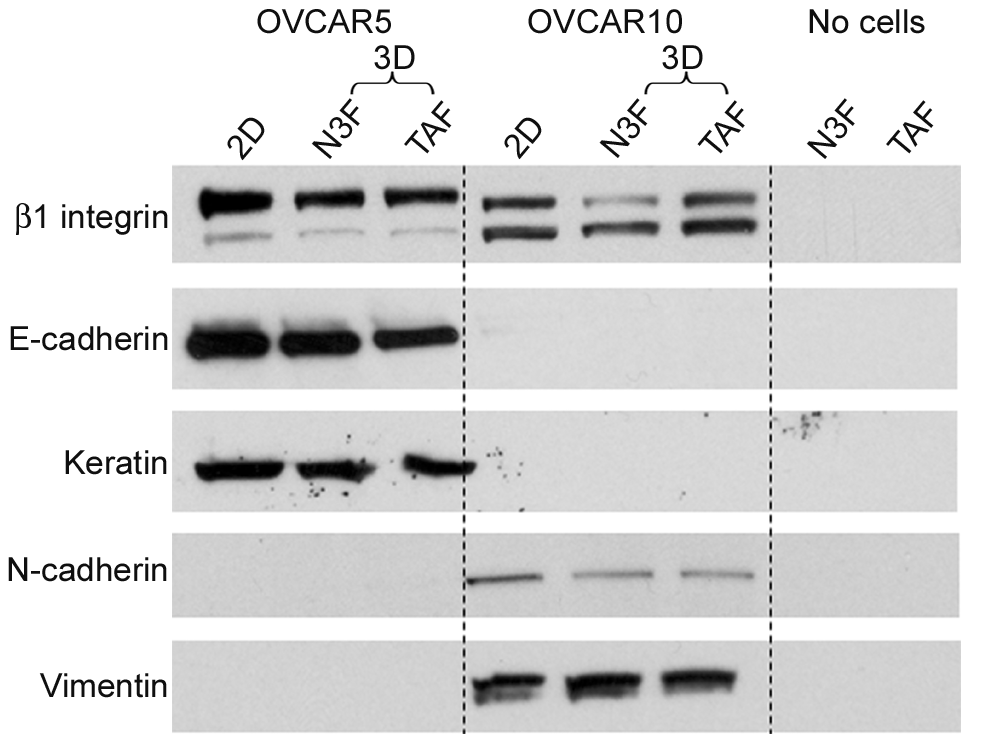

Supplement: Figure S1 — Expression of epithelial and mesenchymal markers in OVCAR5 and OVCAR10 cells grown in 2D and 3D (N3F- and TAF-derived matrices) conditions. Note that matrices (N3F and TAF-derived) did not contribute to appreciable amount of any proteins tested. For cell lysates obtained from 3D cultures, matrices maintained without cells were used as controls to subtract proteins derived from matrices. (TIF) [file pone.0018872.s001.tif]

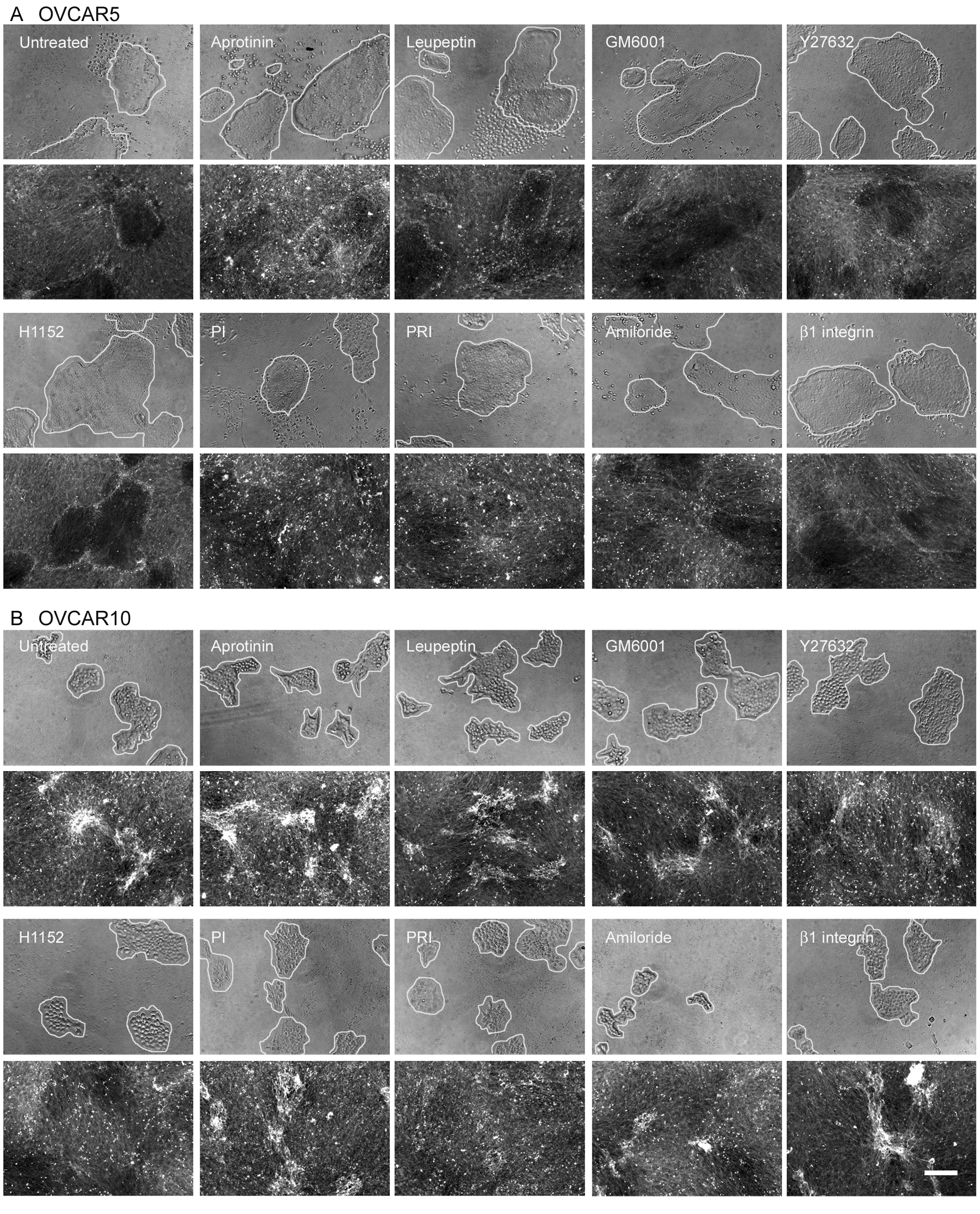

Supplement: Figure S2 — Effect of various inhibitors on matrix remodeling induced by OVCAR5 and OVCAR10 cells. OVCAR5 (A) and OVCAR10 (B) cells were plated onto pre-labeled N3F-derived matrices and cultured under various inhibitory conditions. Phase contrast (cells, top panel) and fluorescence (matrices, bottom panel) images were acquired at 7 days of culture. Bar represents 200 µm. Concentrations of inhibitors used were selected to avoid noticeable inhibition of cell proliferation. PI; a protease inhibitor cocktail of individual protease inhibitors containing aprotinin (7.5 µM), leupeptin (20 µM), and GM6001 (25 µM), PRI; a mixture of PI and H1152 (0.1 µM). (TIF) [file pone.0018872.s002.tif]

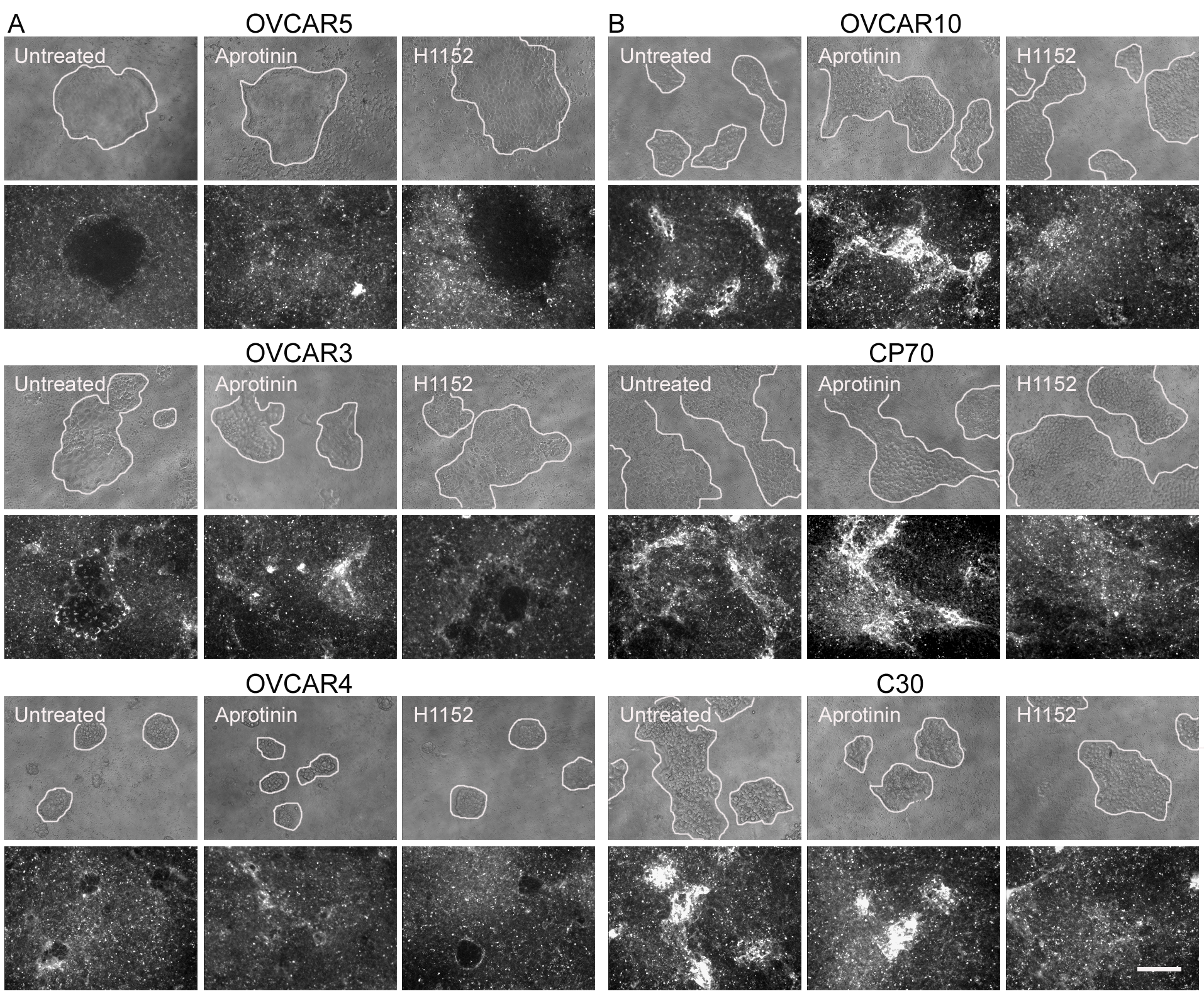

Supplement: Figure S3 — Effect of aprotinin and H1152 on matrix remodeling induced by cells with epithelial and partial EMT phenotypes. OVCAR5-like cells, e.g., OVCAR5, OVCAR3, and OVCAR4 (A), and OVCAR10-like cells, e.g., OVCAR10, CP70, and C30 (B), were plated on pre-labeled N3F-derived matrices and cultured in the absence or presence of aprotinin (7.5 µM) and H1152 (0.1 µM). Phase contrast (cells, top panel) and fluorescence (matrices, bottom panel) images were acquired at 7 days of culture. Bar represents 200 µm. Note that aprotinin effectively inhibited ECM modification induced by OVCAR5, OVCAR3, and OVCAR4 cells which degrade ECMs in contrast to suppression of ECM contraction induced by OVCAR10, CP70, and C30 cells by H1152. (TIF) [file pone.0018872.s003.tif]

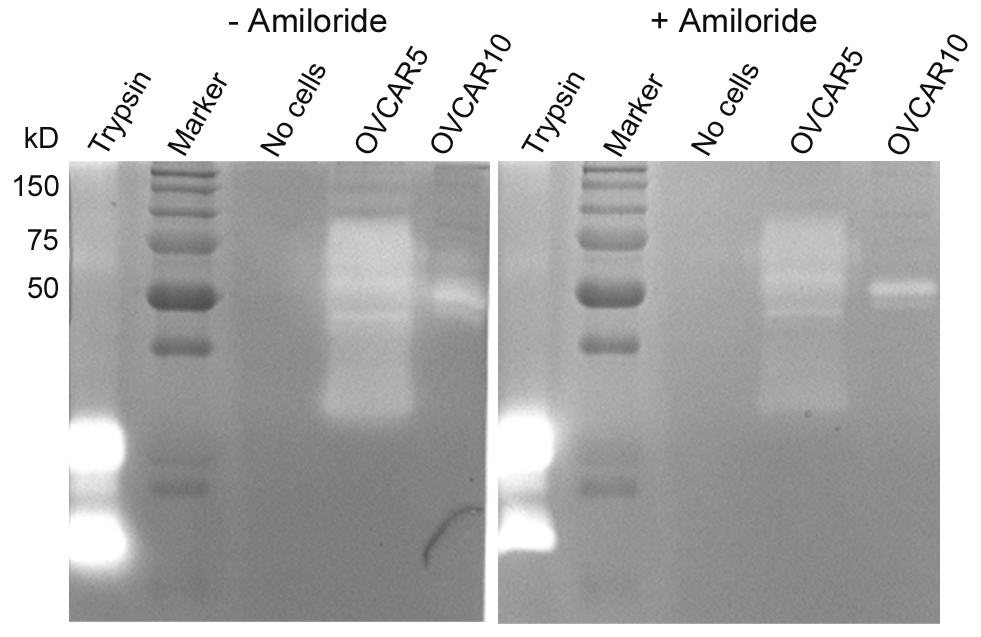

Supplement: Figure S4 — Amiloride on caseinolytic activity derived from OVCAR5 and OVCAR10 cells. Conditioned media derived from 3D (N3F-derived matrices) cultures of OVCAR5 and OVCAR10 cells were subjected to SDS-PAGE using gels copolymerized with casein and plasminogen. Casein gels were incubated with developing buffer for overnight at 37°C in the absence and presence of an uPA inhibitor, amiloride. Note that caseinolytic activity was retained even after the treatment of 1 mM amiloride. (TIF) [file pone.0018872.s004.tif]

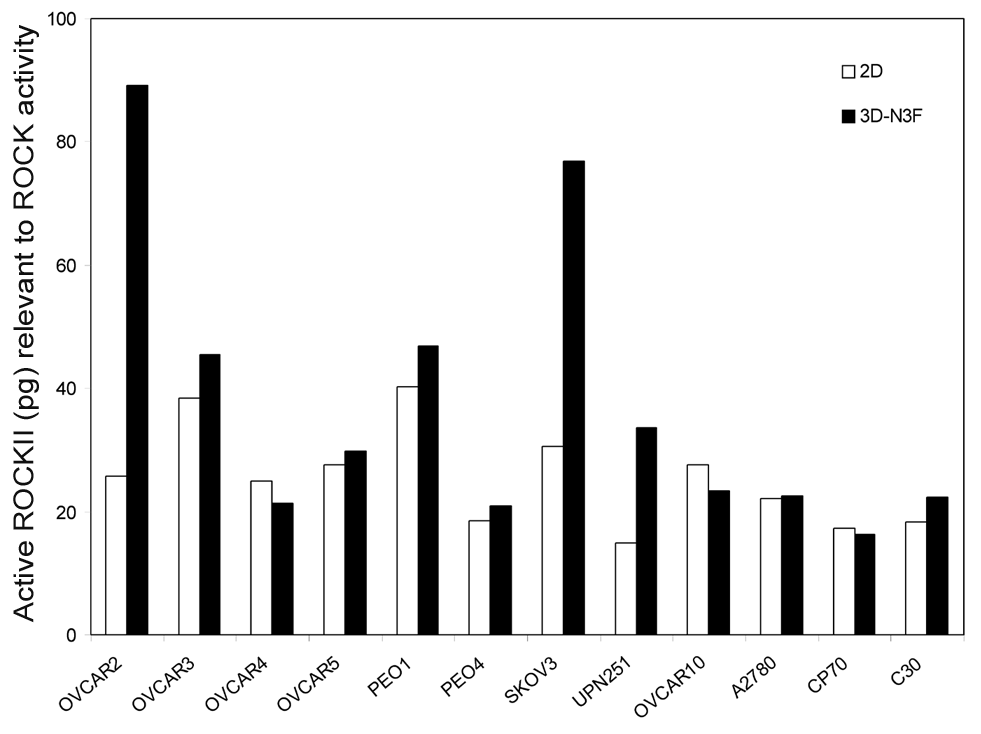

Supplement: Figure S5 — ROCK activity in a panel of ovarian tumor cells. Ovarian tumor cell lysates isolated from cells grown in 2D or 3D (N3F-derived matrices) were subjected to an enzymatic immunoassay using ROCK Activity Assay Kit (see File S1 for details). Cells were grouped according to their ECM remodeling capabilities as shown in Figure 1. ROCK activity was expressed as units (in pg) of purified active ROCKII. (TIF) [file pone.0018872.s005.tif]
