# Supplementary material for: Differential Expressions of Adhesive Molecules and Proteases Define Mechanisms of Ovarian Tumor Cell Matrix Penetration/Invasion
Source: PLoS One. 2011 Apr 19;6(4):e18872. doi: 10.1371/journal.pone.0018872 (PMC3079735; doi:10.1371/journal.pone.0018872)
Supplement: File S1 — Supplemental Methods. (DOC) [file pone.0018872.s006.doc]

# Supplemental Methods

ROCK activity by an enzymatic immunoassay

Cells were grown in 2D and 3D (N3F matrices) until they reached 70% confluence. ROCK activity of cells lysates (30 μg total protein) was analyzed by an enzymatic immunoassay using ROCK Activity Assay Kit (Cell Biolabs, Inc., CA, USA) according to the manufacturer’s protocol. For cell lysates obtained from 3D cultures, lysates of matrices maintained without cells were used as controls to subtract proteins derived from matrices.
