# Supplementary material for: Differential Expressions of Adhesive Molecules and Proteases Define Mechanisms of Ovarian Tumor Cell Matrix Penetration/Invasion
Source: PLoS One. 2011 Apr 19;6(4):e18872. doi: 10.1371/journal.pone.0018872 (PMC3079735; doi:10.1371/journal.pone.0018872)
Supplement: Table S1 — Intensity change of total matrix protein induced by OVCAR5 cells in the presence and absence of various inhibitors measured at the top, middle, and bottom parts of 3D culture. (DOC) [file pone.0018872.s007.doc]

**Table S1.** Intensity change of total matrix protein induced by OVCAR5 cells in the presence and absence of various inhibitors measured at the top, middle, and bottom parts of 3D culture.

| Inhibitors | Top | Middle | Bottom |
| --- | --- | --- | --- |
| Untreated | 43 ± 9.6^bc3)^ | 59 ± 20.1^b^ | 100 ± 21.1^abc^ |
| Y27632 | 45 ± 8.7^bcd^ | 48 ± 9.5^b^ | 92 ± 25.4^ab^ |
| H1152 | 31 ± 7.1^b^ | 52 ± 7.2^b^ | 158 ± 43.6^bcd^ |
| Aprotinin | 112 ± 20.4^de^ | 216 ± 47.6^c^ | 340 ± 66.7^de^ |
| Leupeptin | 105 ± 26.8^cde^ | 104 ± 20.9^bc^ | 152 ± 42.9^abcd^ |
| GM6001 | 51 ± 7.0^bcd^ | 74 ± 15.2^b^ | 142 ± 33.5^abcd^ |
| PI^1)^ | 204 ± 42.7^e^ | 249 ± 93.0^c^ | 325 ± 170.3^cde^ |
| PRI^2)^ | 145 ± 46.3^cde^ | 274 ± 71.0^c^ | 488 ± 113.7^e^ |
| Amiloride | 16 ± 7.2^a^ | 27 ± 11.1^a^ | 54 ± 12.2^a^ |
| β_1_-integrin | 96 ± 30.6^bcde^ | 84 ± 23.1^b^ | 83 ± 17.6^abc^ |

^1)^ PI: protease inhibitor cocktail of aprotinin, leupeptin, and GM6001

^2)^ PRI: cocktail of PI and H1152

^3)^ Mean ± SE (n=5~10), no significant difference (p > 0.01) was found among groups bearing the same letter of alphabets within top, middle, and bottom.
