# Supplementary material for: Differential Expressions of Adhesive Molecules and Proteases Define Mechanisms of Ovarian Tumor Cell Matrix Penetration/Invasion
Source: PLoS One. 2011 Apr 19;6(4):e18872. doi: 10.1371/journal.pone.0018872 (PMC3079735; doi:10.1371/journal.pone.0018872)
Supplement: Table S2 — Intensity change of fibronectin induced by OVCAR5 cells in the presence and absence of various inhibitors measured at the top, middle, and bottom parts of 3D culture. (DOC) [file pone.0018872.s008.doc]

**Table S2.** Intensity change of fibronectin induced by OVCAR5 cells in the presence and absence of various inhibitors measured at the top, middle, and bottom parts of 3D culture.

| Inhibitors | Top | Middle | Bottom |
| --- | --- | --- | --- |
| Untreated | 23 ± 4.5^a3)^ | 41 ± 10.9^ab^ | 100 ± 28.1^ab^ |
| Y27632 | 26 ± 5.5^ab^ | 26 ± 4.9^ab^ | 110 ± 34.0^a^ |
| H1152 | 16 ± 2.8^a^ | 29 ± 4.4^ab^ | 92 ± 28.5^ab^ |
| Aprotinin | 35 ± 14.9^abc^ | 40 ± 23.7^ab^ | 94 ± 59.3^a^ |
| Leupeptin | 29 ± 7.7^ab^ | 36 ± 12.5^ab^ | 80 ± 24.4^a^ |
| GM6001 | 19 ± 2.0^ab^ | 18 ± 2.2^a^ | 30 ± 4.7^a^ |
| PI^1)^ | 77 ± 8.0^c^ | 45 ± 12.8^ab^ | 36 ± 13.4^a^ |
| PRI^2)^ | 23 ± 4.7^ab^ | 60 ± 17.5^ab^ | 159 ± 86.8^ab^ |
| Amiloride | 27 ± 9.7^a^ | 80 ± 27.7^ab^ | 314 ± 60.1^b^ |
| β_1_-integrin | 86 ± 31.6^bc^ | 82 ± 19.1^b^ | 189 ± 53.3^ab^ |

^1)^ PI: protease inhibitor cocktail of aprotinin, leupeptin, and GM6001

^2)^ PRI: cocktail of PI and H1152

^3)^ Mean ± SE (n=5~10), no significant difference (p > 0.01) was found among groups bearing the same letter of alphabets within top, middle, and bottom.
