# Supplementary material for: Differential Expressions of Adhesive Molecules and Proteases Define Mechanisms of Ovarian Tumor Cell Matrix Penetration/Invasion
Source: PLoS One. 2011 Apr 19;6(4):e18872. doi: 10.1371/journal.pone.0018872 (PMC3079735; doi:10.1371/journal.pone.0018872)
Supplement: Table S3 — Intensity change of total matrix protein by OVCAR10 cells in the presence and absence of various inhibitors measured at the top, middle, and bottom parts of 3D culture. (DOC) [file pone.0018872.s009.doc]

**Table S3.** Intensity change of total matrix protein by OVCAR10 cells in the presence and absence of various inhibitors measured at the top, middle, and bottom parts of 3D culture.

| Inhibitors | Top | Middle | Bottom |
| --- | --- | --- | --- |
| Untreated | 186 ± 32.5^de3)^ | 103 ± 16.1^abcd^ | 100 ± 12.3^a^ |
| Y27632 | 92 ± 12.4^bc^ | 80 ± 8.1^abc^ | 98 ± 9.7^a^ |
| H1152 | 58 ± 7.9^ab^ | 65 ± 4.7^ab^ | 90 ± 10.1^a^ |
| Aprotinin | 139 ± 23.5^cd^ | 114 ± 12.7^cd^ | 102 ± 9.7^a^ |
| Leupeptin | 214 ± 19.6^de^ | 149 ± 9.4^d^ | 109 ± 10.7^a^ |
| GM6001 | 245 ± 41.1^de^ | 156 ± 20.2^d^ | 150 ± 34.2^a^ |
| PI^1)^ | 172 ± 38.8^de^ | 118 ± 11.2^cd^ | 110 ± 15.4^a^ |
| PRI^2)^ | 46 ± 10.6^a^ | 63 ± 10.5^a^ | 100 ± 9.7^a^ |
| Amiloride | 258 ± 30.6^e^ | 162 ± 19.0^d^ | 150 ± 24.6^a^ |
| β1-integrin | 186 ± 38.0^de^ | 101 ± 20.1^abcd^ | 82 ± 17.3^a^ |

^1)^ PI: protease inhibitor cocktail of aprotinin, leupeptin, and GM6001

^2)^ PRI: cocktail of PI and H1152

^3)^ Mean ± SE (n=5~10), no significant difference (p > 0.01) was found among groups bearing the same letter of alphabets within top, middle, and bottom.
