# Supplementary material for: Differential Expressions of Adhesive Molecules and Proteases Define Mechanisms of Ovarian Tumor Cell Matrix Penetration/Invasion
Source: PLoS One. 2011 Apr 19;6(4):e18872. doi: 10.1371/journal.pone.0018872 (PMC3079735; doi:10.1371/journal.pone.0018872)
Supplement: Table S4 — Intensity change of fibronectin induced by OVCAR10 cells in the presence and absence of various inhibitors measured at the top, middle, and bottom parts of 3D culture. (DOC) [file pone.0018872.s010.doc]

**Table S4.** Intensity change of fibronectin induced by OVCAR10 cells in the presence and absence of various inhibitors measured at the top, middle, and bottom parts of 3D culture.

| Inhibitors | Top | Middle | Bottom |
| --- | --- | --- | --- |
| Untreated | 1093 ± 187.5^c3)^ | 202 ± 18.8^a^ | 100 ± 22.9^a^ |
| Y27632 | 561 ± 107.6^ab^ | 179 ± 36.2^a^ | 119 ± 26.8^a^ |
| H1152 | 319 ± 65.4^a^ | 137 ± 30.2^a^ | 111 ± 30.0^a^ |
| Aprotinin | 868 ± 165.5^bc^ | 224 ± 33.0^a^ | 111 ± 27.2^a^ |
| Leupeptin | 683 ± 114.2^bc^ | 185 ± 25.1^a^ | 81 ± 13.4^a^ |
| GM6001 | 1019 ± 177.5^bc^ | 230 ± 44.1^a^ | 89 ± 27.5^a^ |
| PI^1)^ | 696 ± 81.2^bc^ | 224 ± 45.3^a^ | 125 ± 43.1^a^ |
| PRI^2)^ | 334 ± 44.6^a^ | 204 ± 32.5^a^ | 305 ± 99.6^a^ |
| Amiloride | 1143 ± 187.7^c^ | 215 ± 29.1^a^ | 113 ± 20.3^a^ |
| β1-integrin | 858 ± 52.3^bc^ | 158 ± 22.6^a^ | 102 ± 19.1^a^ |

^1)^ PI: protease inhibitor cocktail of aprotinin, leupeptin, and GM6001

^2)^ PRI: cocktail of PI and H1152

^3)^ Mean ± SE (n=5~10), no significant difference (p > 0.01) was found among groups bearing the same letter of alphabets within top, middle, and bottom.
